# Aerosol Iron from Metal Production as a Secondary Source of Bioaccessible Iron

**DOI:** 10.1021/acs.est.2c06472

**Published:** 2023-02-28

**Authors:** Akinori Ito, Takuma Miyakawa

**Affiliations:** Yokohama Institute for Earth Sciences, Japan Agency for Marine-Earth Science and Technology (JAMSTEC), 3173-25 Showa-machi, Kanazawa-ku, Yokohama, Kanagawa 236-0001, Japan

**Keywords:** bioaccessible iron, metal production, anthropogenic aerosol, mineral dust, air pollution

## Abstract

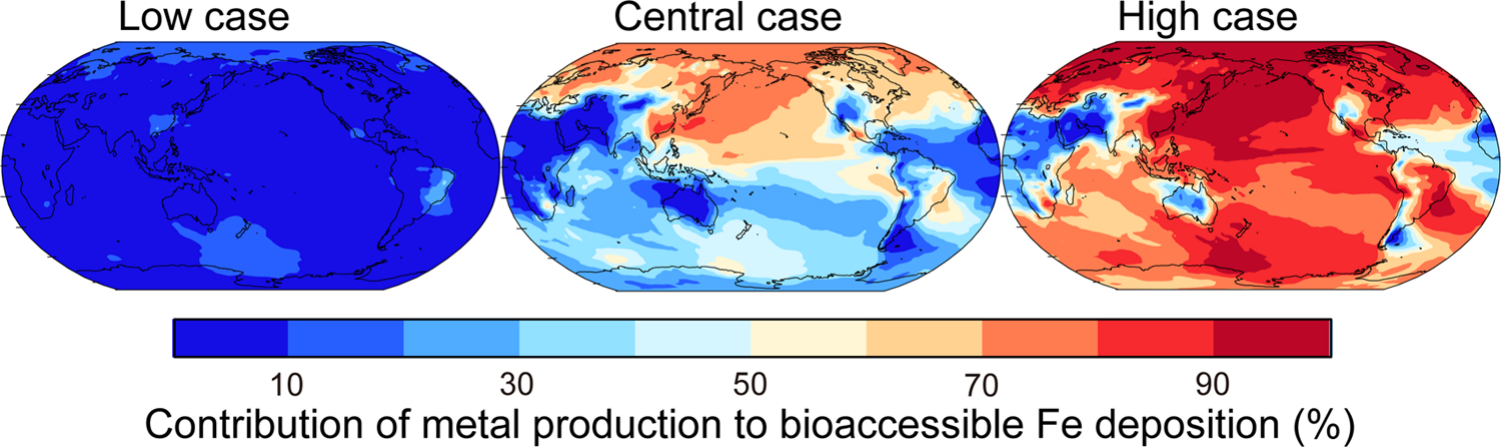

Atmospheric iron (Fe) from anthropogenic, lithogenic, and pyrogenic sources contributes to ocean fertilization, climate change, and human health risk. However, significant uncertainties remain in the source apportionment due to a lack of source-specific evaluation of Fe-laden aerosols. Here, the large uncertainties in the model estimates are investigated using different Fe emissions from metal production. The best agreement in the anthropogenic factor of aerosol Fe concentrations with the field data in the downstream region of East Asian outflow (median: 0.026 μg m^–3^) is obtained with the low case (0.023 μg m^–3^), whereas the best agreement of aerosol Fe bioaccessibility with field data (4.5%) over oceans south of 45°S is obtained with the high case (4.9%). Our simulation with the low case confirms that anthropogenic aerosols play dominant roles in bioaccessible Fe deposition in the northwestern Pacific, compared to lithogenic sources. Our simulations with higher cases suggest that Fe-containing particles co-emitted with sulfur dioxide from metal production substantially contribute to atmospheric bioaccessible Fe fluxes to the Southern Ocean. These findings highlight that accurate representation of aerosol Fe from metal production is a key to reduce large uncertainties in bioaccessible Fe deposition fluxes to the Southern Ocean (0.7–4.4 Gg Fe year^–1^).

## Abstract 2

Iron-containing particles co-emitted with sulfur dioxide from metal production substantially contribute to atmospheric bioaccessible iron fluxes to the Southern Ocean, mainly due to a rapid dissolution and small particle size.

## 1 Introduction

Atmospheric depositions of leachable iron (Fe) from anthropogenic (metal production and fossil fuel combustion), lithogenic (mineral dust), and pyrogenic (open biomass burning) aerosols represent important external sources of micronutrients to the open ocean, which could affect climate through marine biogeochemical feedback.^1,2^ A traditional view is that lithogenic aerosol dominates the atmospheric supply of potentially bioavailable Fe to the world ocean, compared to the anthropogenic and pyrogenic aerosols. Contemporarily, the low-latitude Fe supply from the lithogenic source contributes to nitrogen fixation by diazotrophs, whereas Fe supply from other sources such as continental margin and upwelled hydrothermal sources at the high latitudes regulates the magnitude and dynamics of marine primary productivity.^3^

Bioavailable Fe can be taken up by the ocean biota immediately, whereas bioaccessible Fe is potentially bioavailable, partly because aerosols can be scavenged into sinking materials and be deeply removed from the surface ocean toward the seafloor (i.e., ballasting effect).^4^ Thus, the term bioaccessible Fe is used for water-soluble Fe in aerosols, which is more bioavailable than insoluble forms such as crystalline Fe oxides in soils. Compared to the lithogenic Fe, the anthropogenic and pyrogenic sources co-emit metals and acidic species (i.e., sulfur dioxide (SO_2_) and nitrogen oxides), which enhance the acidity of particulate matter (PM) and bioaccessibility of aerosol Fe (i.e., the fraction of bioaccessible Fe in total aerosol Fe) by orders of magnitude.^5−7^ Indeed, the 2019–2020 Australian wildfires could supply pyrogenic Fe with higher bioaccessibility than lithogenic Fe and trigger widespread phytoplankton growth in high-nutrient, low-chlorophyll (HNLC) regions of the Southern Ocean.^2,8,9^ Meanwhile, individual particle observations confirm that more than 65% of nanosized Fe-containing particles are internally mixed with sulfates and nitrates over eastern China.^10^ Thus, nanoparticulate Fe oxides in anthropogenic aerosols are substantially transformed into bioaccessible Fe under acidic conditions during the aerosol lifetime.^5,11,12^ At the same time, inhalation of Fe and copper (Cu) in PM_2.5_ (particulate matter less than 2.5 μm in diameter) causes a variety of adverse health effects due to the formation of reactive oxygen species through the Fenton reaction.^13^ Although lithogenic aerosol dominates the mass concentration of PM, oxidative potential concentration is associated mostly with anthropogenic sources.^14^ Therefore, accurate quantification of both the emission source strength of aerosol Fe and source apportionment is crucial for the environmental assessment and mitigation policies.

Source apportionment of ambient PM has been extensively performed using a receptor model, which is a statistical analysis of air pollution measurements.^15,16^ The receptor modeling algorithm is essentially a weighted least-squares technique that apportions time-series profiles of specific compounds to the airborne PM mass concentrations.^17^ Since the sources of anthropogenic and lithogenic PM need to be identified for efficient and effective control strategies of air quality management, most previous studies have investigated the source apportionment of PM over urban and industrial areas in the Northern Hemisphere.^18^ Over a megacity in eastern China, the receptor model suggests that industrial emissions contribute less than 20% to PM_2.5_ but are the major contributor to bioaccessible Fe (44–72%).^10^ Consequently, bioaccessible Fe in aerosols from anthropogenic sources are substantially delivered to the northwestern Pacific.^19−21^ At the same time, some countries in the Southern Hemisphere such as Zambia and Chile are heavily polluted by SO_2_ and trace metals from the mining industry.^22,23^ Indeed, ship-based observations over the tropical Pacific have suggested influences of smelting emissions on bioaccessible metal concentrations across the eastern Pacific, with enhanced concentrations near the smelting facilities.^24,25^ However, Fe emission estimates from metal production remain highly uncertain, and its inclusion in the emission inventory could increase anthropogenic Fe source flux in the fine aerosols by an order of magnitude (Table S1^11,26,27^). Smelting sources include iron-ore sintering, pig-iron production, steel-making, aluminum (Al), Cu, lead (Pb), and zinc (Zn) smelting.^27^ Indeed, to match the observed magnetite mass concentration over the middle and high latitudes of the oceans in the Southern Hemisphere, the anthropogenic emission flux in southern Africa^27^ has been multiplied by a factor of 5.^28^

Aerosol transport model simulations have been performed to determine the source contributions of aerosol Fe from anthropogenic, lithogenic, and pyrogenic sources.^11,26−32^ The aerosol Fe concentrations and their bioaccessibilities from four models have been comprehensively evaluated with field data during multiple research cruises, but significant differences in the source contribution among the model estimates exist.^6,32^ To validate the representations of anthropogenic, lithogenic, and pyrogenic Fe in aerosol transport models, time-series measurements of air quality at atmospheric monitoring stations are valuable tools due to the episodic nature of dust storms and wildfires.^2,33^ Furthermore, highly time-resolved monitoring data of trace metals in PM_2.5_ have been used to evaluate the model performance over the megacities of eastern China^34^ and over western Japan in the downstream region of East Asian outflow.^35,36^ However, direct association of model estimates to total Fe observation can introduce biases in the relative contribution of different sources of aerosol Fe when the observational data are used as a reference for mixed air masses from different sources. On the other hand, isotope-based observations have been used to identify anthropogenic Fe as an isotopically light Fe source and verify the anthropogenic contribution to marine aerosol Fe in aerosol transport models.^21,37^ However, the total emission flux is not well constrained by Fe stable isotope data alone.

Here, we combine high-time-resolution field observations and an aerosol Fe model to verify the source apportionment of aerosol Fe. We focus on the model validation of aerosol Fe emissions from metal production using observed concentrations at Fukue island in the downstream region of East Asian outflow. We isolate the model bias in aerosol Fe concentration to anthropogenic and lithogenic factors with 4-hourly data sets of black carbon (BC) and silicon (Si), respectively. We examine the contributions of aerosol Fe from a metal production source as one of the major uncertainties in anthropogenic Fe emissions.

## 2 Methods

We examine the uncertainties in anthropogenic Fe emissions using four different numerical experiments that varied from zero, low, central, and high estimates of smelting Fe emissions based on a statistical database and four different emission factors of Fe for metal smelting^27^ (Figure S1 and Table S1). In Section 2.1, we describe the observational data set, which is used to evaluate the anthropogenic and lithogenic factors of aerosol Fe concentrations. In Section 2.2, we describe the atmospheric chemical transport model, which is used to estimate aerosol Fe concentrations from anthropogenic and lithogenic sources. In Section 2.3, we describe the detailed method to estimate the anthropogenic and lithogenic factors of aerosol Fe concentrations.

### 2.1 Continuous Observational Data Sets of Trace Metals and Black Carbon in Japan

A continuous 4-hourly averaged data set of PM_2.5_ at the Fukue atmospheric environment observatory (32.75°N, 128.68°E, 75 m above sea level) was used for the evaluation of the simulated trace metal concentrations.^38^ The observatory is located on a remote Fukue Island (326 km^2^) in western Japan, receiving continental outflow air masses frequently in the winter–spring season, with negligible influence from local emissions on the island (more than 10 km away from the main township). The measurement period was from March to May 2018. A continuous particulate monitor with X-ray fluorescence (PX-375, Horiba, Ltd., Kyoto, Japan) was used to measure the mass and element concentrations of aerosols. A PM_2.5_ cyclone (URG-2000-30EH, URG Corp.) was used for the PX-375 inlet. Typical air sampling volumes for one cycle of the collection and X-ray analysis were around 4 m^3^ (16.7 L min^–1^ for 4 h). PX-375 provided 4-hourly mass concentrations of the metals in PM_2.5_, including Fe, Si, calcium (Ca), Pb, Cu, manganese (Mn), potassium (K), chlorine (Cl), and sulfur (S). The limit of detection (LOD) was calculated by taking 3 times the standard deviation (3σ) of the intensity of a blank filter (Fe: 1.57 ng m^–3^ and Si: 8.50 ng m^–3^).

The continuous hourly data set of BC concentrations at the Fukue observatory was obtained from observational records using a multi-angle absorption photometer (MAAP, model 5012; Thermo Scientific, Waltham, MA).^39^ The systematic and random uncertainties were estimated as ±14 and ±17%, respectively (±22% in total).^39^ The hourly measurements of BC concentrations were averaged to 4-hourly time resolution, using the arithmetic mean.

### 2.2 Atmospheric Chemical Transport Model

This study used the Integrated Massively Parallel Atmospheric Chemical Transport (IMPACT) model.^40^ The model simulations were performed using a horizontal resolution of 2.0° × 2.5° for latitude by longitude and 47 vertical layers. The simulations were conducted for comparison with the field measurements over Fukue island in 2018. The chemical transport model was driven by the Modern-Era Retrospective analysis for Research and Applications 2 (MERRA-2) reanalysis meteorological data from the National Aeronautics and Space Administration (NASA) Global Modeling and Assimilation Office (GMAO).^41^ The model simulated the emissions, chemistry, transport, and deposition of aerosols and their precursor gases for anthropogenic, pyrogenic, lithogenic, oceanic (sea spray), and biogenic (terrestrial and marine biomes) sources. Anthropogenic and pyrogenic sources were prescribed at emission, whereas lithogenic, oceanic, and biogenic emissions were dynamically simulated. Atmospheric processing from anthropogenic, pyrogenic, lithogenic, and oceanic sources was projected for four distinct aerosol size bins (<1.26, 1.26–2.5, 2.5–5, and 5–20 μm of diameter). The anthropogenic Fe emission and dissolution scheme has been used in Earth system models.^42−44^ This study used the updated version of the Fe dissolution scheme for anthropogenic and pyrogenic sources^12^ and the emission rates for anthropogenic and lithogenic sources. The implementation of the Fe dissolution scheme led to enhancement of bioaccessibility from 0% at emission by faster Fe release at the initial stage with a higher content of magnetite.^12^

We updated the emission data set from anthropogenic sources, following the revised emission data set of the Community Emission Data System (CEDS).^45^ The fine particulate matter emissions from anthropogenic combustion sources were estimated using BC emissions and the fraction of BC in PM_2.5_ for each country, sector, and time.^45^ The super-micron PM emissions were estimated using PM_2.5_ emissions and the fraction of submicron PM in super-micron PM for each sector^11^ except metal production.^27^ The metal content of aerosols except Fe in the iron and steel industry, shipping, and mineral dust was obtained from the compilation of source-specific measurements in PM_2.5_ and coarse particulate matter (PM_10_).^35,46^ In the model simulations, the Fe content in PM from the iron and steel industry and shipping was taken from the default model.^11^ The parameters used to estimate emission fluxes of trace metals are presented in the Supporting Information (Table S2).

For anthropogenic Fe emissions, metal production has been suggested as a dominant source, but the estimate of this flux remains highly uncertain.^27^ The CEDS data set does not include BC emission from manufacturing processes such as the production of iron and steel, aluminum, and other nonferrous metals, which are grouped together as an aggregate as a “metal production sector”.^45,47^ Here, smelting Fe emissions were estimated from the statistical database and emission factors of Fe for metal smelting.^27^ Ferrous and nonferrous metal-related production data were obtained from the Minerals Yearbooks^48^ and the Steel Statistical Yearbooks.^49^ Spatial distribution and monthly variation were imposed by matching Fe emissions for each country to SO_2_ emissions from the CEDS data set.^45,47^ Uncertainty calculations in the Fe emissions were performed using low, central, and high estimates of smelting Fe emission factors.^27^ In an additional sensitivity simulation, the anthropogenic Fe emission from metal production was not included. Thus, the differences between the simulations with and without Fe emission from metal production represent total and bioaccessible Fe from metal production only.

For the mineral content in soils, the model used the mineralogical database.^50,51^ The Fe content in each mineral from the compilation of measurements^50^ is presented in the Supporting Information (Table S3). The mineral fractions in clay- (<2 μm) and silt-sized (between 2 and 63 μm) soils were distributed in the four size bins following the brittle fragmentation theory.^51,52^ All of the Fe-containing minerals were included in the clay-sized soils, while only three minerals (i.e., goethite, chlorite, and feldspars) were in the silt-sized soils.^50^ The data coverage of the mineralogical database for East Asia is not satisfactory as much of the information for this region is compiled in Chinese language publications.^50^ Indeed, the Fe content is relatively enriched in Asian dust (5.27 ± 0.25%) compared to the global average.^53^ Thus, the regionally averaged Fe content (35–50°N, 70–120°E) for each Fe species in clay-sized soils was scaled to that in the clay-sized fraction of Chinese desert sediments.^54^ The scaling factors of the Fe content, which were used to estimate lithogenic emissions of Fe from clay-sized soils in the simulations, are presented in the Supporting Information (Table S4). The comparison of aerosol emission rates from the lithogenic source with previous studies^11,29−31^ is presented in the Supporting Information (Table S5).

### 2.3 Calculation of Anthropogenic and Lithogenic Factors

The anthropogenic and lithogenic factors were calculated using field measurements of Fe-laden PM_2.5_ species at the Fukue observational site. The pyrogenic source was a minor contributor to the total Fe concentration at the observational site during spring and thus was not considered in this case. The linear fitting function expresses time-resolved (*t*) aerosol concentration data as the sum of contributions from source profiles. The optimization process seeks to estimate the relative contribution of aerosol Fe source strength from *j* source processes, *f*_*j*_, of total *M* source processes by comparing time-resolved Fe concentrations, *c*_*t*_, to those of Fe concentrations from *j* source processes, *x*_*j,t*_, based on the assumption in the model that
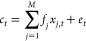
1where *e*_*t*_ represents the residual Fe concentration. The fitting to observed Fe data could be achieved with the tagged tracers of anthropogenic Fe and lithogenic Fe. The inverse modeling technique is successfully applied to the optimization of emission estimates when a major contribution to total Fe concentrations is primarily associated with a single source.^8^

As for mixed air masses from different sources, however, the relative contribution is not well constrained by total Fe concentrations alone. Total Fe data at the observational site showed a positively skewed distribution (skewness = 3.4) mainly due to dust transport episodes. Such high values could introduce biases in the relative source strengths when extremely high concentrations at the peak timing in the observation are not captured by the model simulation. Meanwhile, the time-series profiles might be a useful indicator to estimate the degree to which changes in independent source strengths (predictor) cause changes in a dependent variable when the peak timing in the dependent variable is retained with source profiles by the predictor variables. For this purpose, the regression model was formulated using two elements of Fe-laden aerosols originated from two independent sources. In our case, the predictor variables were trace elements for anthropogenic and lithogenic sources, and the dependent variable was the total Fe concentration. Only one element for each source was selected because the introduction of highly correlated variables into a regression model tends to increase the uncertainty in the estimated regression parameters.^55^ The selection criteria were that the predictor variables should vary independently of one another and that they be primarily associated with a major source.

The model results indicated that BC (median: 96%), Pb (99%), and Cu (97%) in PM_2.5_ were predominantly influenced by anthropogenic sources at the observational site during spring, whereas Si (median: 86%) was predominantly influenced by lithogenic sources (Figure S2). Thus, significantly positive relationships were estimated between anthropogenic BC and sum of anthropogenic and pyrogenic BC in the model estimates (Kendall rank correlation coefficient: *r* = 0.93), as well as between lithogenic Si and the sum of lithogenic, anthropogenic, and pyrogenic Si in the model estimates (*r* = 0.94). A correlation analysis of the observational data yielded significantly positive relationships between BC and Pb (*r* = 0.65), as well as between BC and Cu (*r* = 0.55). The results confirmed that these three elements were strongly influenced by anthropogenic sources. In contrast, a weaker relationship was observed between BC and Si (*r* = 0.36) than between Pb and Si (*r* = 0.43) and between Cu and Si (*r* = 0.48). Thus, the use of Pb or Cu instead of BC could result from the anthropogenic factor mixed with the lithogenic factor. Accordingly, eq 1 could be modified to
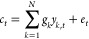
2where *g*_*k*_ represents the relative contribution of source-specific, *k*, aerosol concentrations, *y*_*k,t*_, from total *N* source processes, e.g., BC and Si from anthropogenic (*g*_BC,anthropogenic_ = 0.13 ± 0.01) and lithogenic (*g*_Si,lithogenic_ = 0.339 ± 0.003) factors for observations, respectively (adjusted *R*^2^ = 0.97). The measured data below the LOD for *k* and μ species were replaced with half of the LOD. The missing data were eliminated for the statistical analysis when one of the measurements for *k* and μ species was missing at the same time. The resulting total number of data points was 544. BC and Si concentrations in PM_2.5_ were used to fit the function (2) to Fe concentrations for the field observations. The values of *g*_*k*_*y*_*k*_ were used to evaluate the source fluxes from the anthropogenic and lithogenic factors in the model. The aerosol Fe concentrations fitted from anthropogenic and lithogenic factors were in good agreement with the observed Fe concentrations (Figure S3). Thus, we conclude that quite good fits to the data were achieved for this data set.

## 3 Results and Discussion

Anthropogenic Fe emission estimates from metal production are highly uncertain. Our estimate of anthropogenic Fe emission with the central estimate of smelting Fe emission factors on a global scale (1.1 Tg Fe year^–1^ in submicron PM for 2018) was consistent with a previous estimate from the central case^27^ (1.1 Tg Fe year^–1^ for 2010) (Table S1). Meanwhile, our estimates clearly indicated large emission fluxes from Zambia and northern Chile in the Southern Hemisphere (Figure S1). Here, the impact of the large uncertainties in anthropogenic emissions is explored using four different Fe emission factors for metal production.

We evaluate our results from the sensitivity simulations against aerosol Fe concentrations and aerosol Fe bioaccessibility from field data in Section 3.1. We confirm the dominant contribution of aerosol Fe from anthropogenic sources over the northwestern Pacific in the downstream region of East Asian outflow (Section 3.2). To elucidate the differences in bioaccessible Fe deposition in the Southern Ocean between different simulations, the results from the uncertainty calculations in the smelting Fe emission factors are compared with previous modeling studies (Section 3.3).

### 3.1 Evaluation of Aerosol Fe Concentrations at the Continuous Observational Site

The 4-hourly averaged data set of aerosol BC, Si, and Fe concentrations in PM_2.5_ at Fukue station in western Japan was used for the evaluation of the simulations. The model estimate of BC (0.40 ± 0.32 μg m^–3^, median: 0.31 μg m^–3^) was in good agreement with the observation (0.27 ± 0.23 μg m^–3^, median: 0.20 μg m^–3^) (Figure S2). The model estimate of Si (0.89 ± 1.4 μg m^–3^, median: 0.47 μg m^–3^) was also in good agreement with the observations of Si (0.81 ± 1.2 μg m^–3^, median: 0.48 μg m^–3^). The median of aerosol Fe concentration from field data at Fukue (0.17 μg m^–3^) during spring in 2018 was in the middle of that with the low estimate (0.14 μg m^–3^) of smelting Fe emission factors and that with the central estimate (0.19 μg m^–3^) (Figure S4).

**Figure 1:**
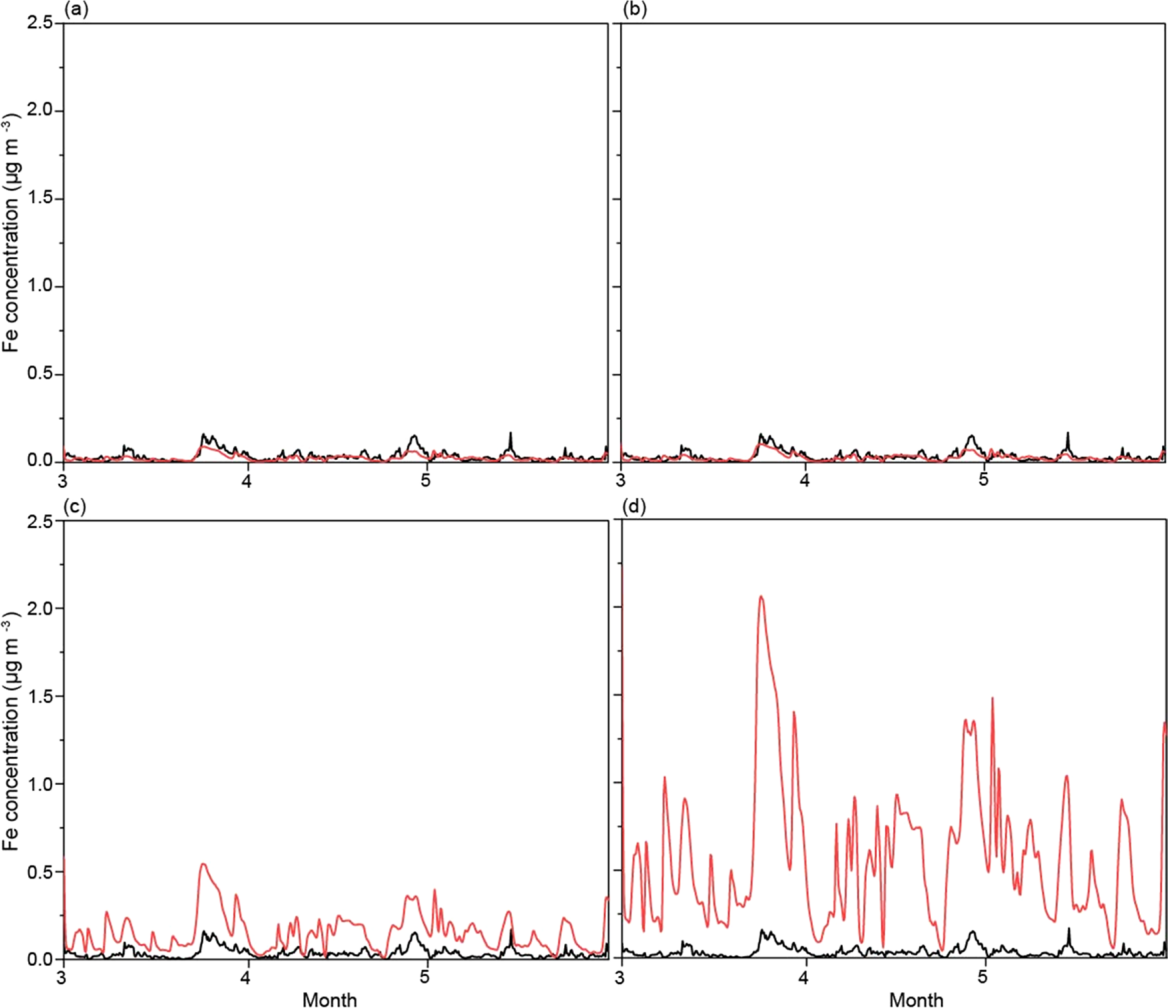
Comparison of the anthropogenic Fe factor in PM_2.5_ (μg m^–3^) from the model estimate (red line) with field data (black line) at Fukue. Uncertainty calculations in the smelting Fe emission factors were performed using (a) zero, (b) low, (c) central, and (d) high estimates of smelting Fe emission factors.^27^

We evaluated our results of the source contributions from the model simulations against field data in the downstream region of East Asian outflow (Figure 1). The simulation with the low estimate of smelting Fe emission factors showed the best agreement of the anthropogenic Fe factor (0.027 ± 0.020 μg m^–3^, median: 0.023 μg m^–3^) with field data (0.035 ± 0.030 μg m^–3^, median: 0.026 μg m^–3^), compared to that with the central estimate of smelting Fe emission factors (0.077 ± 0.065 μg m^–3^, median: 0.060 μg m^–3^). The results of the best agreement of the anthropogenic factor at the Fukue observational site with the low estimate of smelting Fe emission factors were robust, compared to other simulations, even when Pb or Cu was used as an anthropogenic tracer (Figure S5). The results demonstrated that high-time-resolution measurements of source-specific tracers offered model validation for source apportionment of total Fe originated from anthropogenic sources.

**Figure 2:**
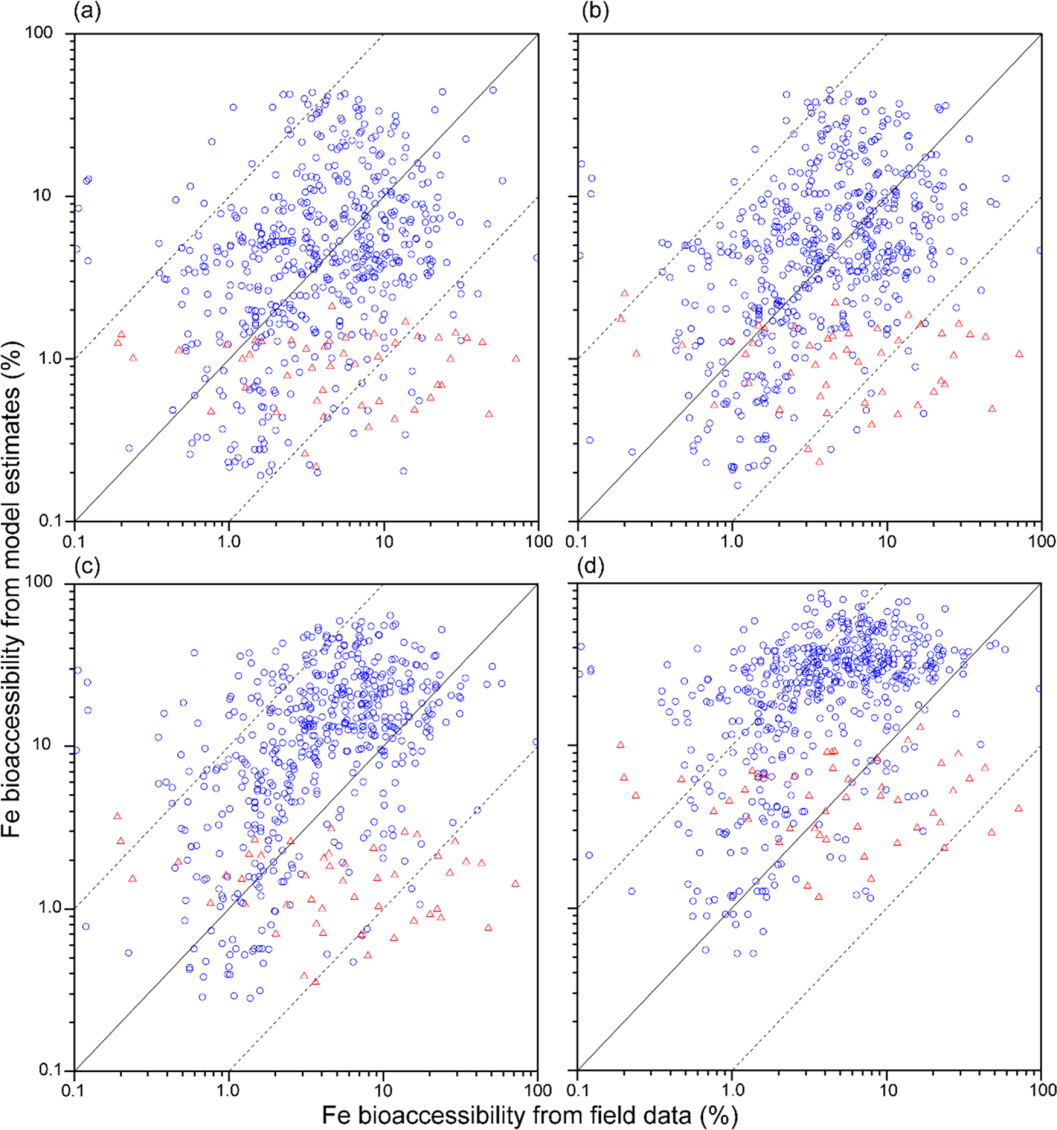
Comparison of aerosol Fe bioaccessibility with field data over oceans south of 45°S and other oceanic regions. Only the simulated data for which maximum likelihood estimates of total Fe concentrations fall within ±2σ_0_ of the measurements were used for the comparison with field data.^1,6^ The numbers of data points were 49 and 539 in oceans south of 45°S (red squares) and other oceanic regions (blue circles), respectively. Uncertainty calculations in the smelting Fe emission factors were performed using (a) zero, (b) low, (c) central, and (d) high estimates of smelting Fe emission factors.^27^ The solid line represents a 1-to-1 correspondence. The dashed lines show deviations from the solid line by a factor of ±10.

Since the Fe emission factors could vary by smelting facilities in different countries, we evaluated the model prediction of aerosol Fe bioaccessibility on a global scale (Figure 2). We separated the evaluation of aerosol Fe bioaccessibility over oceans south of 45°S and the rest of the global ocean because the former was the region for which models underestimated aerosol Fe bioaccessibility.^6^ The simulation with the higher estimates of smelting Fe emission factors indicated better agreement of aerosol Fe bioaccessibility (median: 4.9% for the high case) with field data (4.5%) over oceans south of 45°S. On the other hand, the simulation with the lower estimates of smelting Fe emission factors showed better agreement (4.7% for the low case) with field data (3.7%) over the rest of the global ocean. These results suggested that improvement of modeled Fe bioaccessibility required higher estimates of smelting Fe emission factors for southern countries than others.

### 3.2 Contribution of Aerosol Fe from Anthropogenic and Lithogenic Sources

**Figure 3:**
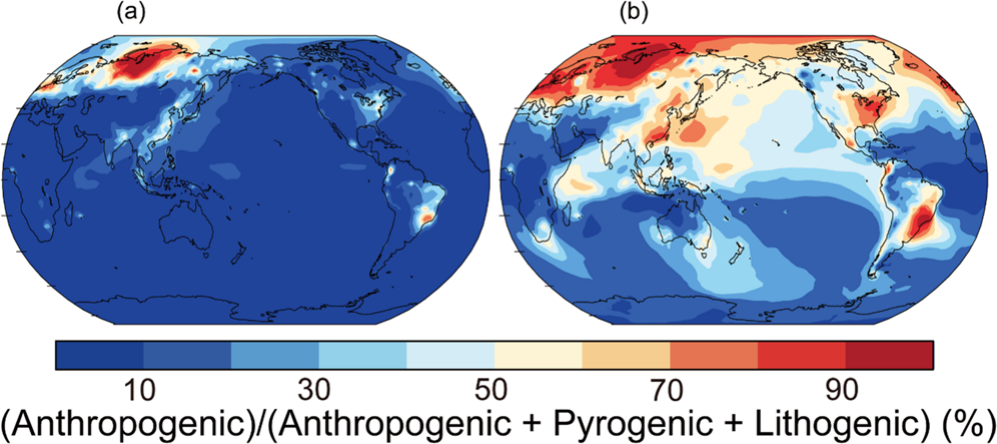
Percentage contribution of the anthropogenic source to the (a) total and (b) bioaccessible Fe concentration in PM_2.5_ (%) from the simulation with the low estimate of smelting Fe emission factors near the ground surface in 2018. The color represents the (anthropogenic)/(anthropogenic + pyrogenic + lithogenic) ratio (%).

Anthropogenic Fe contributed to 0.5–82% (median: 18%) of 4-hourly Fe concentration in fine particles at the observational site after the inclusion of the low estimate of smelting Fe emission factors (Figure S4). On the other hand, lithogenic and pyrogenic sources contributed to 14–100% (median: 81%) and 0.0–16.0% (median: 0.5%), respectively. The annually averaged total Fe concentrations in PM_2.5_ with the low estimate of smelting Fe emission factors indicated that anthropogenic Fe contributed to less than 20% over the northwestern Pacific (Figure 3a). The higher values were estimated over the northwestern Pacific closer to East Asia. These results were consistent with Fe isotope-based observations even when the smelting Fe emission was not considered^21^ (Figure S6a). The simulation with the low estimate of smelting Fe emission factors suggested that anthropogenic emissions contributed to more than 60% of bioaccessible Fe in PM_2.5_ over eastern China (Figure 3b). The model result of the major contributor of the anthropogenic source to bioaccessible Fe was consistent with the receptor model in the Hangzhou megacity (30°N, 120°E).^10^

**Figure 4:**
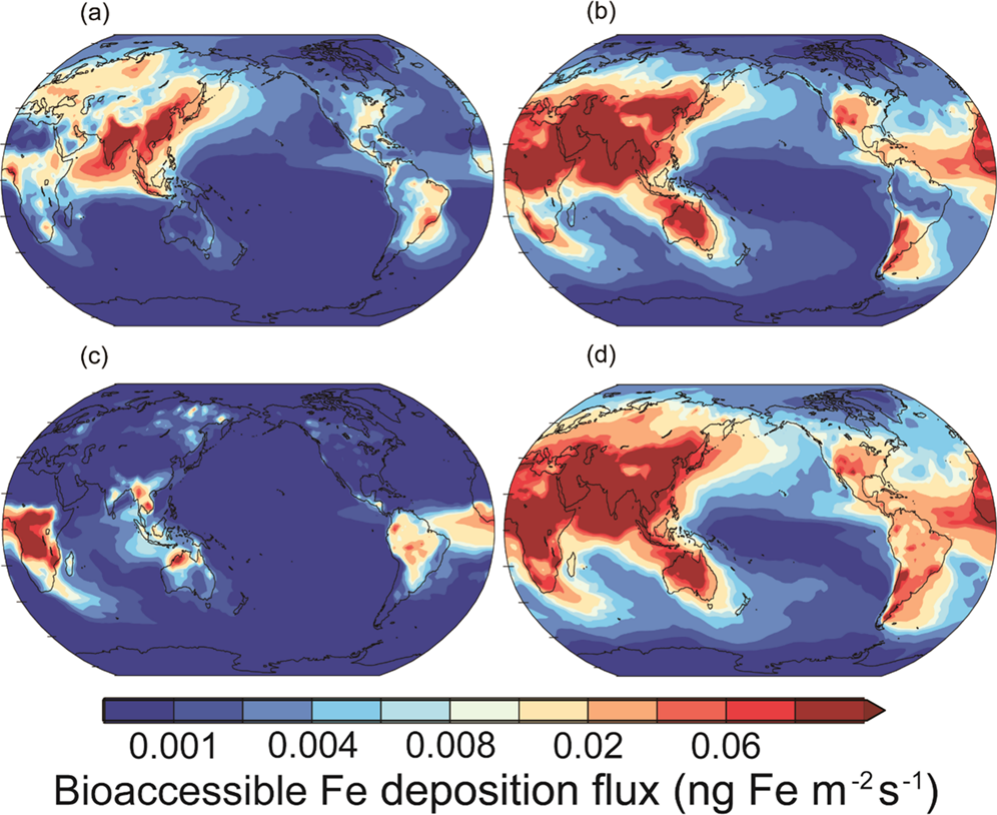
Deposition fluxes of bioaccessible Fe (ng Fe m^–2^ s^–1^) from (a) anthropogenic (33 and 0.12 Gg Fe year^–1^), (b) lithogenic (168 and 0.52 Gg Fe year^–1^), (c) pyrogenic (15 and 0.09 Gg Fe year^–1^), and (d) the sum of the three sources to oceans (216 and 0.72 Gg Fe year^–1^) from the simulation with the low estimate of smelting Fe emission factors. The parentheses represent bioaccessible Fe deposition fluxes to the global ocean and the Southern Ocean (>60°S), respectively.

Our results with the low estimate of smelting Fe emission factors confirmed that the anthropogenic source was the major contributor of bioaccessible Fe deposition fluxes to the northwestern Pacific, compared to lithogenic and pyrogenic sources (Figure S7). The bioaccessible Fe deposition flux from the anthropogenic source to the Southern Ocean (0.12 Gg Fe year^–1^) was comparable to the pyrogenic source (0.09 Gg Fe year^–1^) (Figure 4). However, a lack of laboratory experiments for pyrogenic aerosols hinders the accurate representation of aerosol Fe bioaccessibility. Thus, it is desirable to develop the Fe dissolution scheme for pyrogenic aerosols to reduce the uncertainty in model estimates.

### 3.3 Secondary Source of Bioaccessible Fe from Metal Production

**Figure 5:**
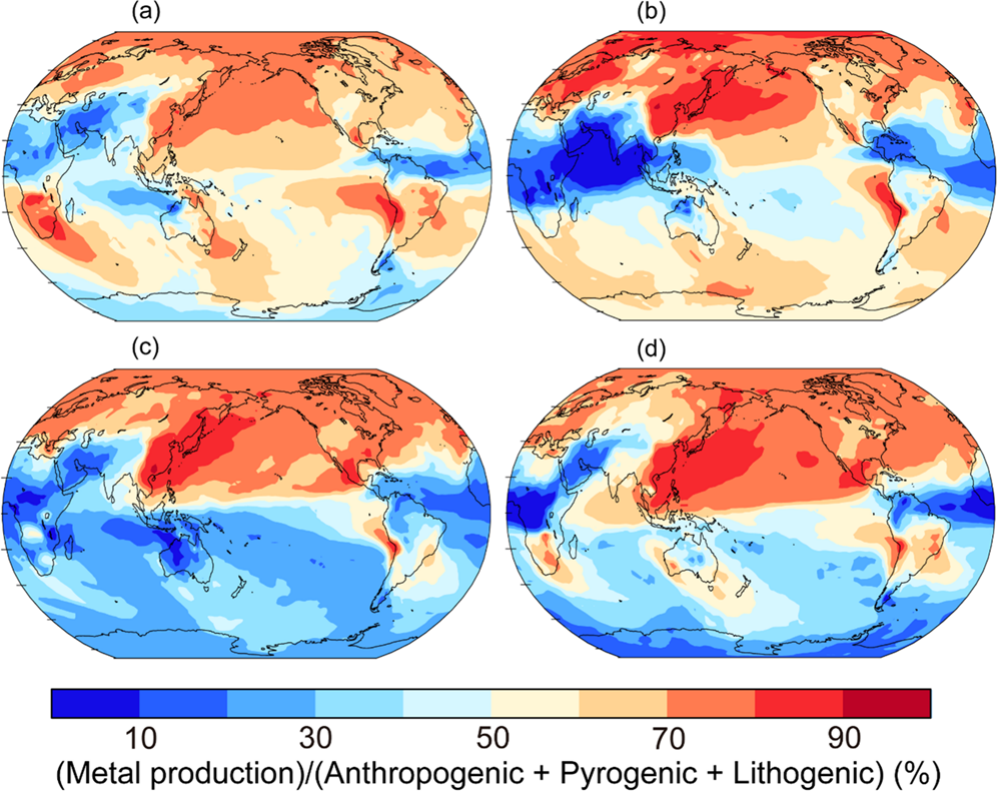
Percentage contribution of metal production to bioaccessible Fe deposition fluxes (%) for (a) austral fall (50 and 45%), (b) winter (18 and 58%), (c) spring (41 and 28%), and (d) summer (72 and 35%) from the simulation with the central estimate of smelting Fe emission factors (austral fall: March, April, and May; winter: June, July, and August; spring: September, October, and November; summer: December, January, and February). The color represents the (metal production)/(anthropogenic + pyrogenic + lithogenic) ratio (%). The parentheses represent averaged contribution of metal production with the central estimate to the global ocean and the Southern Ocean (>60°S), respectively.

Our bioaccessible Fe deposition flux with the low estimate of smelting Fe emission factors from combustion aerosols (i.e., the sum of anthropogenic and pyrogenic sources except for metal production) to the Southern Ocean (0.15 Gg Fe year^–1^) was comparable to previous estimates (0.06–0.19 Gg Fe year^–1^) (Table S6). Since the models underestimate bioaccessible Fe concentrations over oceans south of 45°S compared to field observations by a factor of 15,^6^ the impact of the large uncertainties on anthropogenic emissions was explored using the higher estimates of smelting Fe emission factors, compared to the low case (Figure S8). Our simulations with the central estimate of smelting Fe emission factors suggested that Fe-containing particles co-emitted with SO_2_ from metal production could supply the major secondary source of bioaccessible Fe in aerosol to the Southern Ocean (58%) in austral winter (Figure 5). The contribution of metal production to bioaccessible Fe deposition fluxes indicated a strong seasonality due to dust and wildfire activities. Furthermore, the sum of bioaccessible Fe deposition flux to the Southern Ocean with the high estimate of smelting Fe emission factors (4.4 Gg Fe year^–1^) was considerably higher than previous estimates (0.17–0.51 Gg Fe year^–1^)^11,29−31^ (Figure S9 and Table S6).

Our estimate of bioaccessible Fe deposition flux to the Earth’s surface with the central estimate of smelting Fe emission factors (1.05 Tg Fe year^–1^) was substantially lower than a previous estimate (1.96 Tg Fe year^–1^)^27^ whereas that to the southern region (>60°S) (1.26 Gg Fe year^–1^) was considerably higher than the previous estimate (0.53 Gg Fe year^–1^) (Table 1). Our model indicated significantly larger estimates of total and bioaccessible Fe deposition fluxes from high-latitude dust into the southern region (194 Gg Fe year^–1^ and 0.62 Gg Fe year^–1^) than the previous estimates (14 Gg Fe year^–1^ and 0.27 Gg Fe year^–1^). Moreover, Fe bioaccessibilities from anthropogenic sources were substantially enhanced during atmospheric transport to the southern region (>60°S) in our model (33–44%), compared to the global values (9.1–22%). In contrast, the previous study assumes the same dissolution rates of magnetite Fe from anthropogenic sources as lithogenic, resulting in much lower Fe bioaccessibilities for anthropogenic Fe (2.6%) but higher for lithogenic Fe (2.0%),^27^ compared to our estimates (33–44 and 0.3%, respectively). These results reflected faster Fe dissolution rates from anthropogenic sources than from lithogenic sources in our model, based on laboratory experiments.^12^

**Table 1:** Comparison of Total and Bioaccessible Fe Deposition Fluxes to the Earth’s Surface and Southern Region between This Study and the Previous Study^27^^a^

	total Fe				bioaccessible Fe		
study	anthropogenic	pyrogenic		lithogenic	anthropogenic	pyrogenic	lithogenic
Earth’s surface (Tg Fe year^–1^)							
low case	1.5	0.5	97		0.10 (9.1%)	0.06 (12%)	0.57 (0.6%)
central case	2.7				0.42 (16%)		
high case	7.4				0.16 (22%)		
ref (27)	2.2	2.3	131		0.04 (1.9%)	0.23 (10%)	1.69 (1.3%)
southern region (>60°S) (Gg Fe year^–1^)							
low case	0.40	0.36	194		0.13 (33%)	0.10 (28%)	0.62 (0.3%)
central case	1.3				0.54 (42%)		
high case	9.5				4.28 (44%)		
ref (27)	0.44	1.6	14		0.01 (2.6%)	0.25 (16%)	0.27 (2.0%)

a Note: uncertainty calculations in the smelting Fe emission factors were performed using low, central, and high estimates of smelting Fe emission factors.^27^ Parentheses represent fractional Fe bioaccessibilities (%).

## 4 Implication of Aerosol Fe from Metal Production on Marine Biogeochemistry and Phytoplankton Productivity

Air pollution from metal smelting emissions has been reported for some countries in the Southern Hemisphere. The use of an atmospheric Fe model provides valuable information on where and how much atmospheric deposition fluxes from smelting Fe emissions may be important. The global model simulations reveal where intensive field observations are required to better understand the processes affecting smelting Fe emissions to improve the emission inventory and bioaccessible Fe deposition flux in the marine receptor region. Our global model results with the higher estimates of smelting Fe emission factors suggested a substantial supply of bioaccessible Fe from southern Africa to the southwestern Indian Ocean, as well as from southern America to the tropical south-east Pacific. Meanwhile, ship-based observations have suggested that elevated concentrations of bioaccessible Cu, Fe, cobalt (Co), and nickel (Ni) over the tropical south-east Pacific offshore of Peru are most likely related to emissions from metal smelting facilities in the south of Peru or northern Chile.^24^ Additionally, Fe isotope analysis indicates that both particulate and dissolved Fe at a water depth above 50 m along the Peruvian coasts are isotopically lighter than atmospheric mineral dust.^56^ The light isotopic signatures might support the dominant roles of anthropogenic aerosols in bioaccessible Fe deposition to the tropical south-east Pacific with the higher estimates of smelting Fe emission factors. However, this hypothesis requires careful evaluation of other Fe sources and isotope fractionation during phytoplankton uptake.^57^

The southeastern region of Madagascar exhibits a major sporadic phytoplankton bloom, the South-East Madagascar Bloom, in austral summer and fall.^58^ The South-East Madagascar Bloom in winter is a recurrent and regular phenomenon of the phytoplankton phenology but is weaker than in summer.^59^ Atmospheric deposition of aerosol Fe from industrial sources in southern Africa has been hypothesized to enhance marine biological productivity in the South Indian Ocean.^60^ Thus, the high percentage fluxes of bioaccessible Fe from metal production to the southwestern Indian Ocean in austral summer and fall might be associated with the South-East Madagascar Bloom. This possible bioaccessible Fe from anthropogenic sources and its implication on the growth of phytoplankton, the fishery, and food security in southern African countries shall be addressed in a future study. There are, however, large uncertainties in the effects of anthropogenic and pyrogenic fluxes on the Fe cycling in the oceans, partly because most previous studies focus on lithogenic sources, which could shorten the residence time of fine particles from anthropogenic sources in models due to the ballasting effects.^42,61^ Additionally, positive feedback between biological production and Fe bioavailability might sustain the dissolved Fe in the surface ocean and prolong the phytoplankton response.^62,63^

The sensitivity results highlight the importance of laboratory experiments for aerosols from the metal production industry in atmospheric Fe models. Future work should focus on the high-time-resolution measurements of trace elements from smelting emissions in regions near the source and downstream of the source, simultaneously. This may help in understanding the ecological effects on marine biomes, as well as the possible adverse health on the communities close to the smelting facilities. The framework developed here should be applicable to other elements for mixed air masses from different primary sources. This is especially crucial for assessing the impact of air quality on the ecosystem, climate, and human health.

## Supporting Information Available

Comparison of anthropogenic Fe emission fluxes (Figure S1); comparison of simulated BC, Si, Pb, and Cu concentrations (Figure S2); comparison of aerosol Fe concentrations from observations in PM_2.5_ with fitting model estimates at Fukue in spring (Figure S3); comparison of source contribution of aerosol Fe concentrations in PM_2.5_ between model simulations with field data at Fukue in spring (Figure S4); comparison of anthropogenic Fe factor from model simulations with three different smelting Fe emission factors in PM_2.5_ with field data at Fukue in spring (Figure S5); percentage contribution of anthropogenic source to the total and bioaccessible Fe concentration in PM_2.5_ (Figure S6); percentage contribution of bioaccessible Fe deposition fluxes from anthropogenic, lithogenic, and pyrogenic (Figure S7); percentage contribution of metal production to bioaccessible Fe deposition fluxes for austral fall, winter, spring, and summer (Figure S8); and percentage contribution of metal production to bioaccessible Fe deposition fluxes for austral fall, winter, spring, and summer (Figure S9) Annual Fe emissions from anthropogenic sources in different global studies (Table S1); summary of the metal content of aerosols (Table S2); summary of Fe content in each mineral (Table S3); summary of scaling factors of Fe content (Table S4); annual aerosol emissions from lithogenic sources and averaged Fe content by weight (Table S5); and comparison of bioaccessible Fe deposition flux (Gg Fe year^–1^) to the world ocean and the Southern Ocean between this study and previous studies (Table S6) (PDF)
