# Supplementary material for: Aerosol Iron from Metal Production as a Secondary Source of Bioaccessible Iron
Source: Environ Sci Technol. 2023 Feb 28;57(10):4091–100. doi: 10.1021/acs.est.2c06472 (PMC10018757; doi:10.1021/acs.est.2c06472)
Supplement: Supplementary file 1 — es2c06472_si_001.pdf [file es2c06472_si_001.pdf]

Supporting Information

Aerosol iron from metal production as a secondary source of bioaccessible iron

Akinori Ito [a, *] and Takuma Miyakawa [a]

[a] Yokohama Institute for Earth Sciences, Japan Agency for Marine-Earth Science and Technology (JAMSTEC), 3173-25 Showa-machi, Kanazawa-ku, Yokohama, Kanagawa 236-0001, Japan.

Corresponding Author

Akinori Ito (akinorii@jamstec.go.jp)

Supporting information: figures and tables that describe additional model details, sensitivity simulations, and comparison of model outputs with previous studies.

- Figures S1 to S9
- Tables S1 to S6

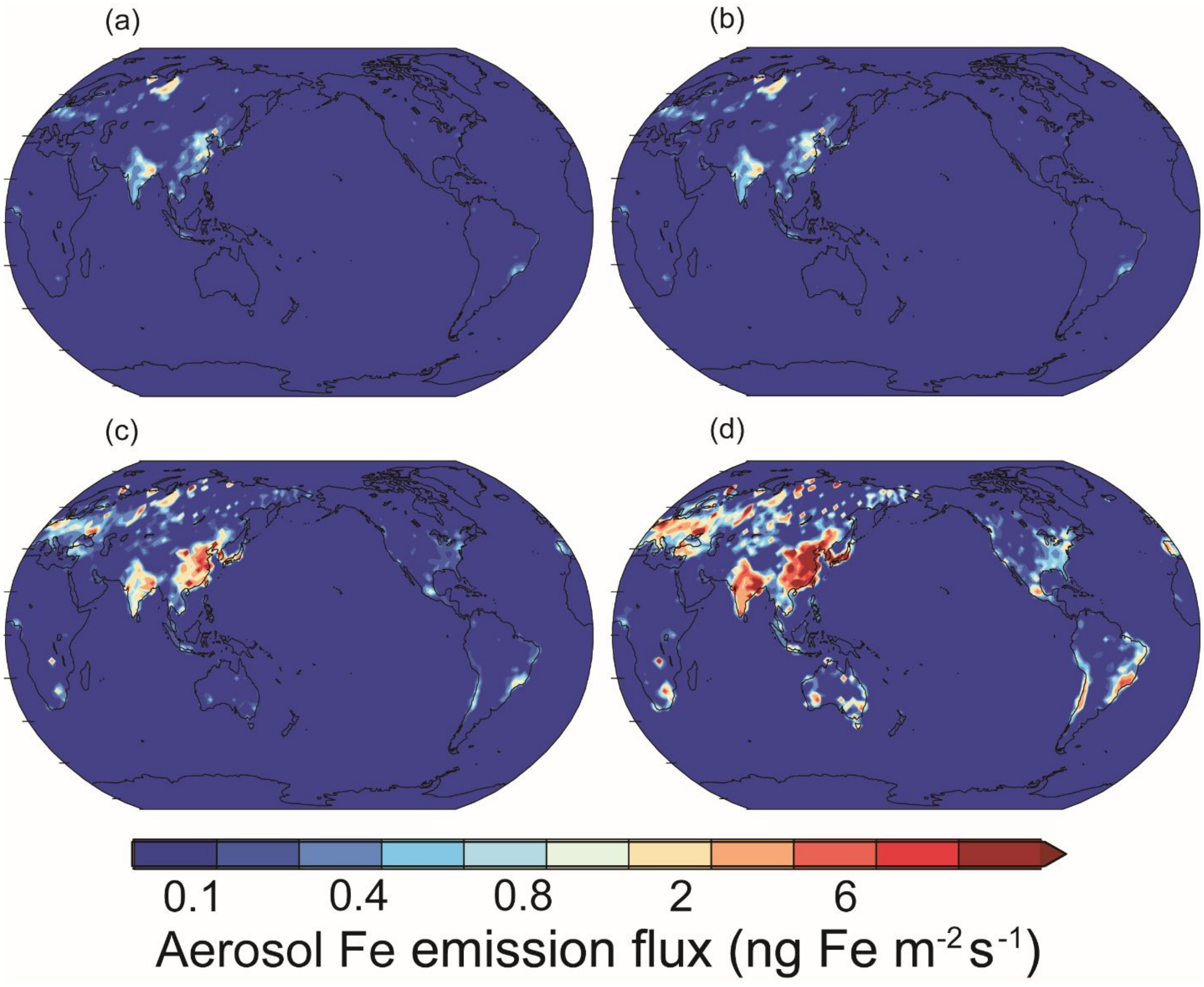

Figure S1. Comparison of anthropogenic Fe emission fluxes (ng m^{-2} s^{-1}) in fine aerosol size (smaller than 1 μm) from four different smelting Fe emission cases. Uncertainty calculations in the smelting Fe emission factors were performed using (a) zero, (b) low, (c) central, and (d) high estimates of smelting Fe emission factors [27].

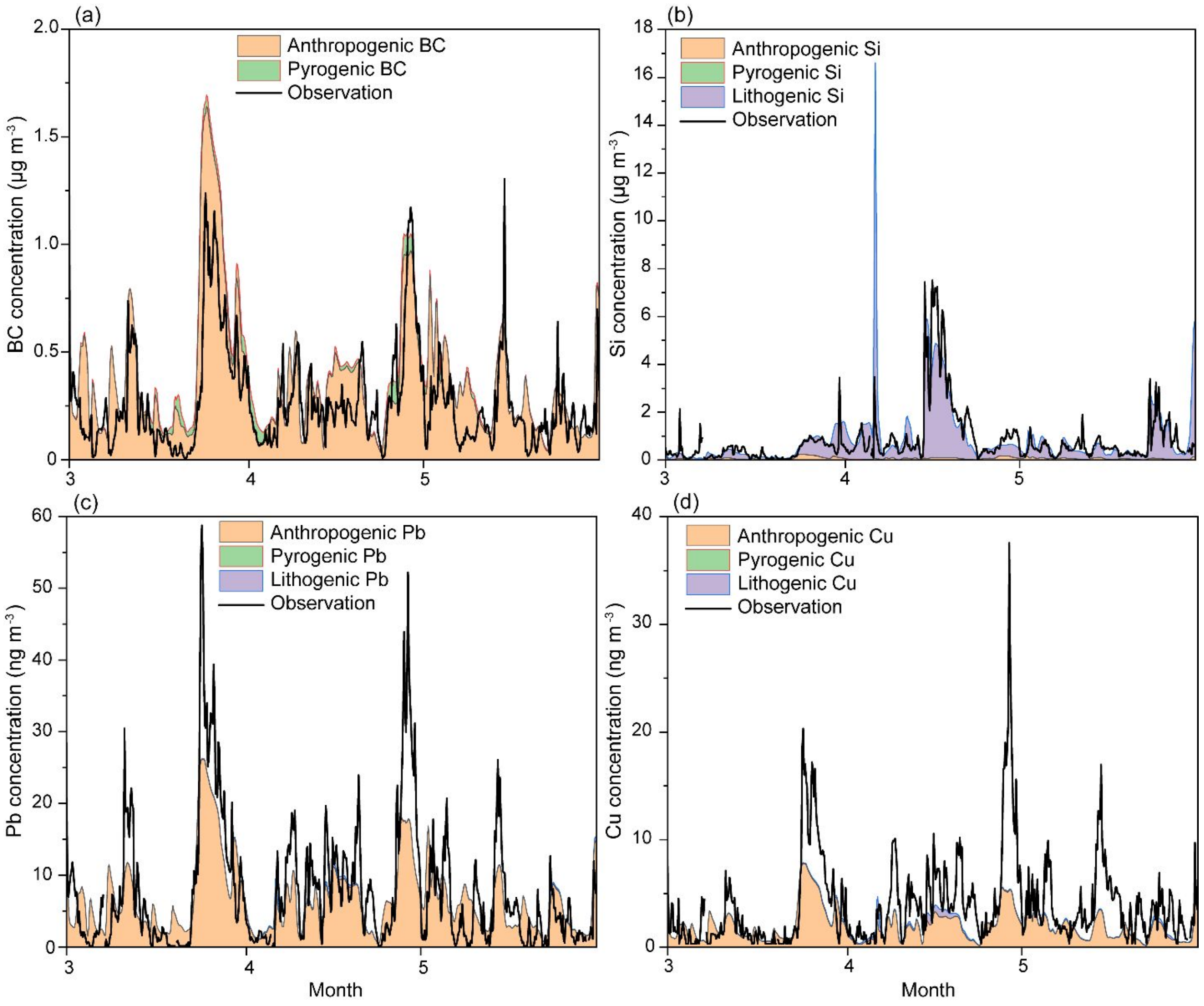

Figure S2. Comparison of simulated (a) BC, (b) Si, (c) Pb, and (d) Cu concentrations (μg m^{-3}) in $PM_{2.5}$ with field data (black line) at Fukue in spring. The color represents the contribution from anthropogenic, pyrogenic, and lithogenic sources, which were estimated by using the tagged tracer of each source.

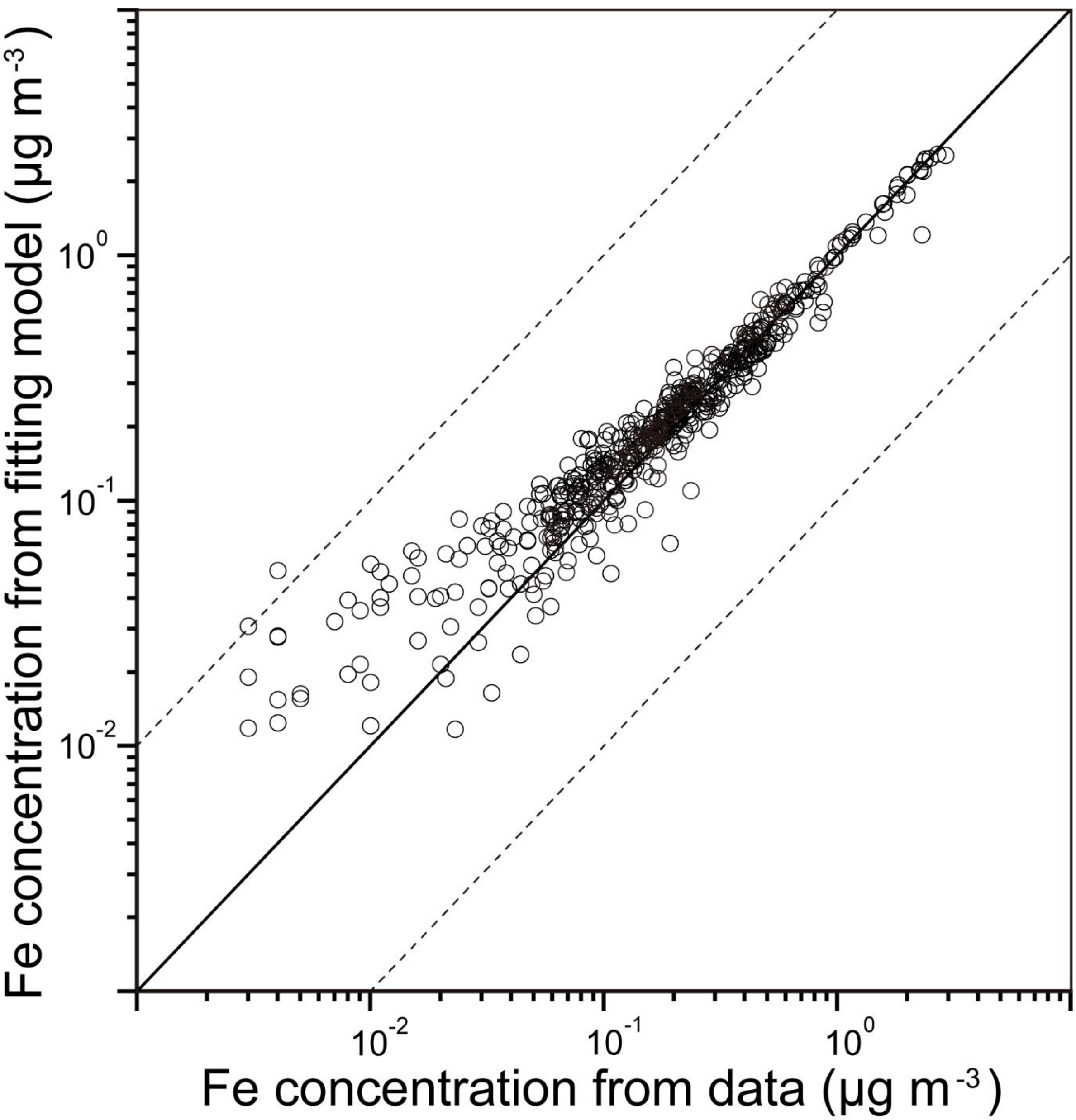

Figure S3. Comparison of aerosol Fe concentrations from observations (μg m^{-3}) in $PM_{2.5}$ with fitting model estimates using equation (2) at Fukue in spring. The solid line represents a 1-to-1 correspondence. The dashed lines show deviations from the solid line by a factor of ±10.

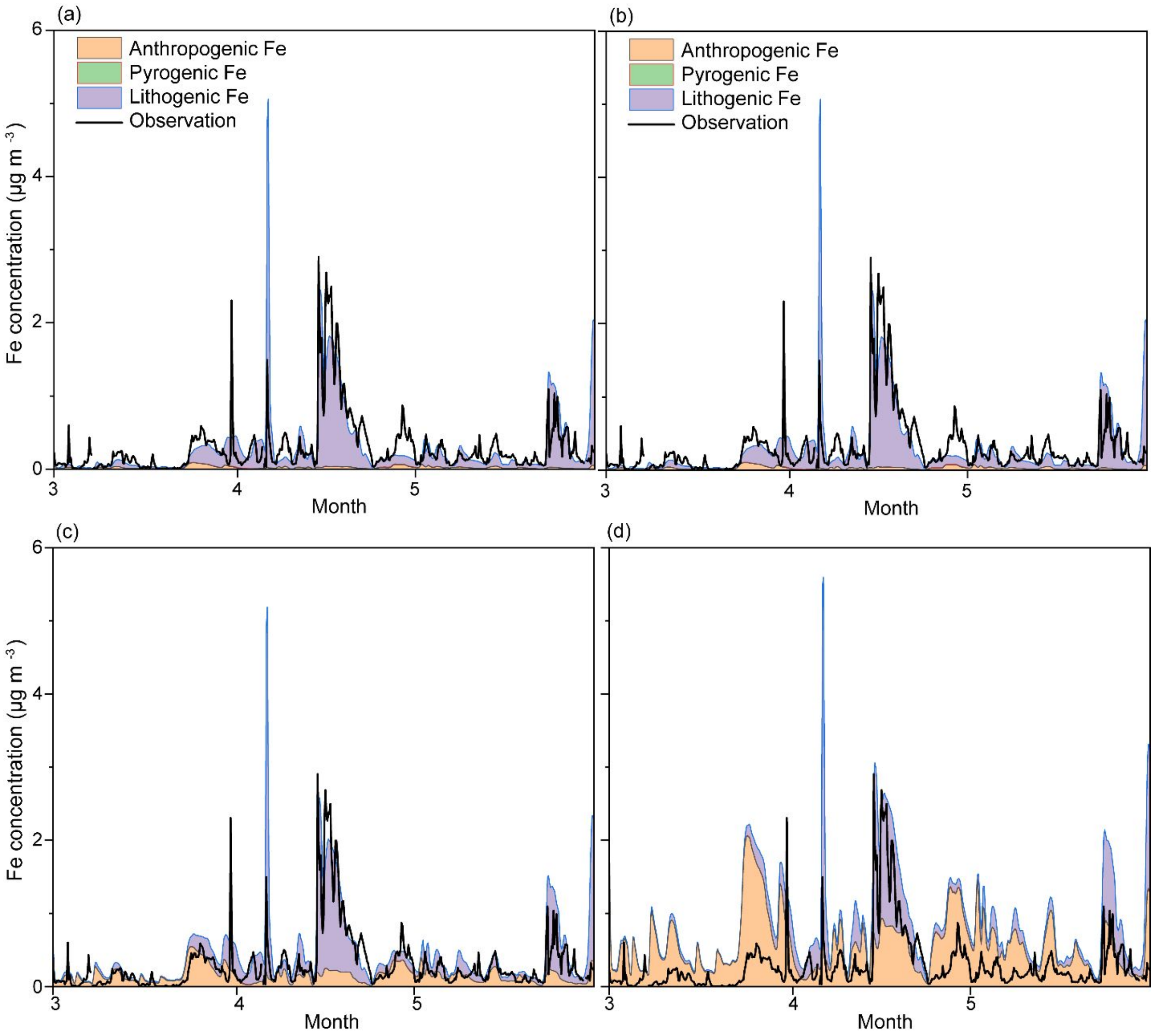

Figure S4. Comparison of source contribution of aerosol Fe concentrations (μg m^{-3}) in $PM_{2.5}$ between model simulations with field data at Fukue in spring. Uncertainty calculations in the smelting Fe emission factors were performed using (a) zero, (b) low, (c) central, and (d) high estimates of smelting Fe emission factors [27]. The color represents the contribution from anthropogenic, pyrogenic, and lithogenic Fe sources, which were estimated by using the tagged tracer of each source. The black line denotes total aerosol Fe concentrations from field data.

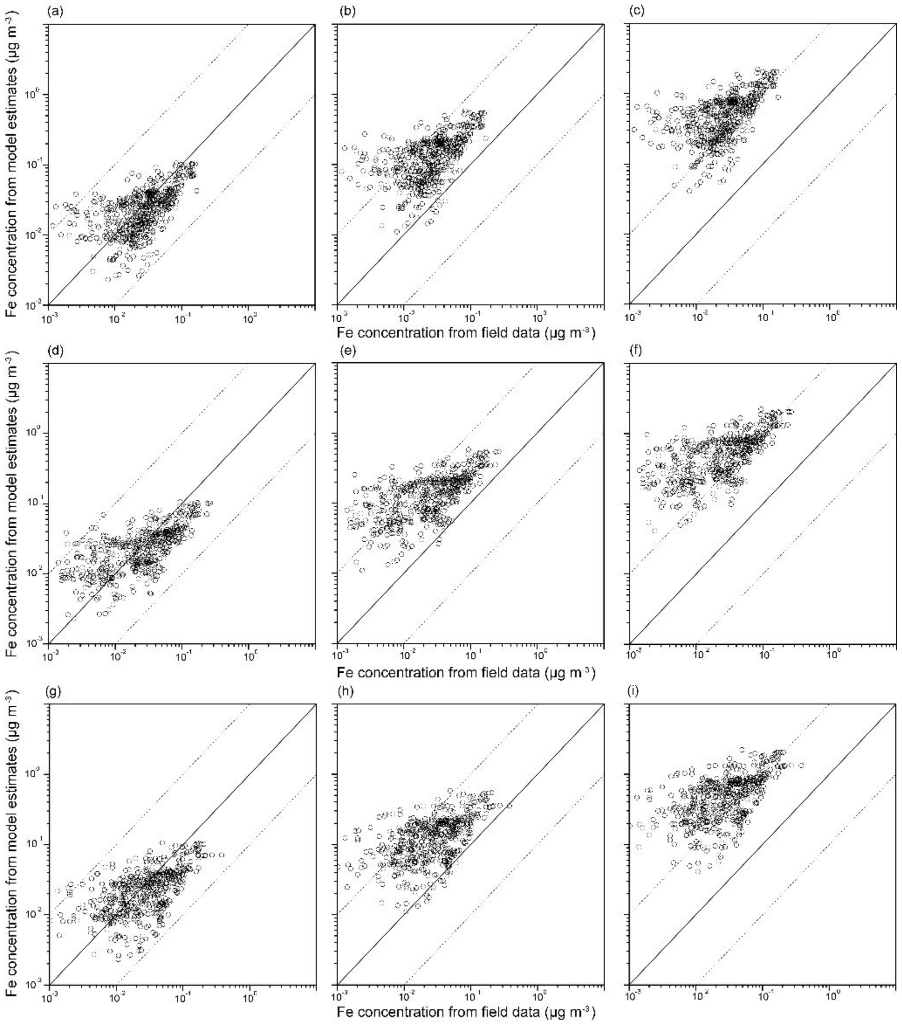

Figure S5. Comparison of anthropogenic Fe factor from model simulations with three different smelting Fe emission factors (μg m^{-3}) in $PM_{2.5}$ with field data at Fukue in spring. Uncertainty calculations in the smelting Fe emission factors were performed using (a) low case from BC and Si, (b) central case from BC and Si, and (c) high case from BC and Si of smelting Fe emission factors [27]. The anthropogenic tracer of Pb, or Cu and lithogenic tracer of Si were used to estimate anthropogenic Fe factor by using equation (2) for (d) low case from Pb and Si, (e) central case from Pb and Si, (f) high case from Pb and Si, (g) low case from Cu and Si, (h) central case from Cu and Si, and (i) high case from Cu and Si. The solid line represents a 1-to-1 correspondence. The dashed lines show deviations from the solid line by a factor of ±10.

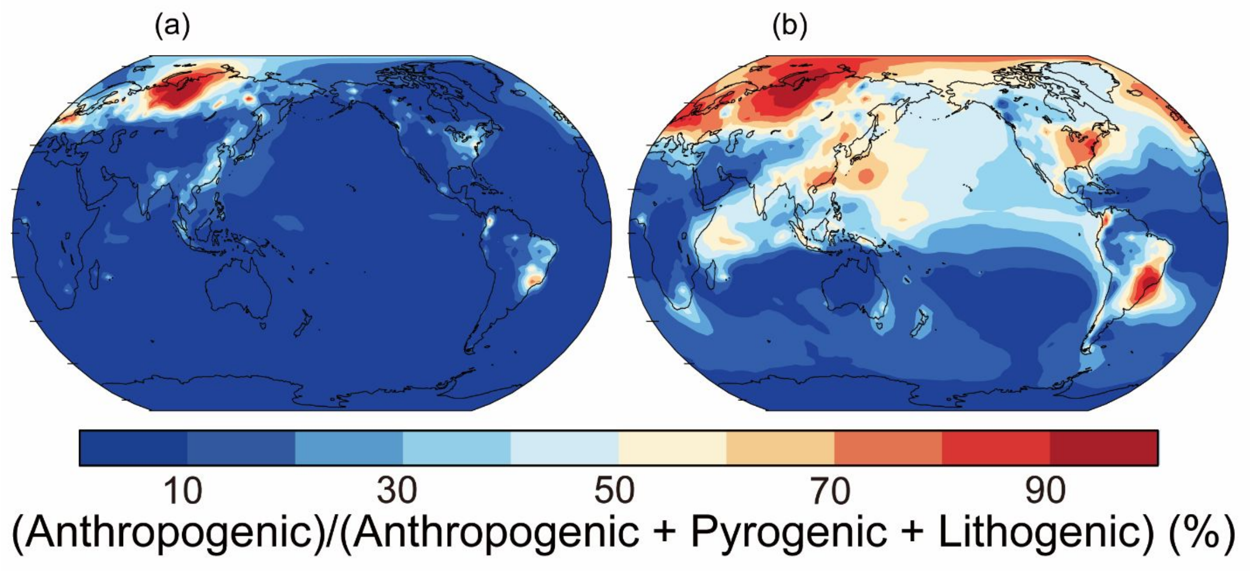

Figure S6. Percentage contribution of anthropogenic source to (a) total and (b) bioaccessible Fe concentration in $PM_{2.5}$ (%) from the simulation with zero smelting Fe emission near the ground surface in 2018. The color represents (anthropogenic)/(anthropogenic + pyrogenic + lithogenic) ratio (percent).

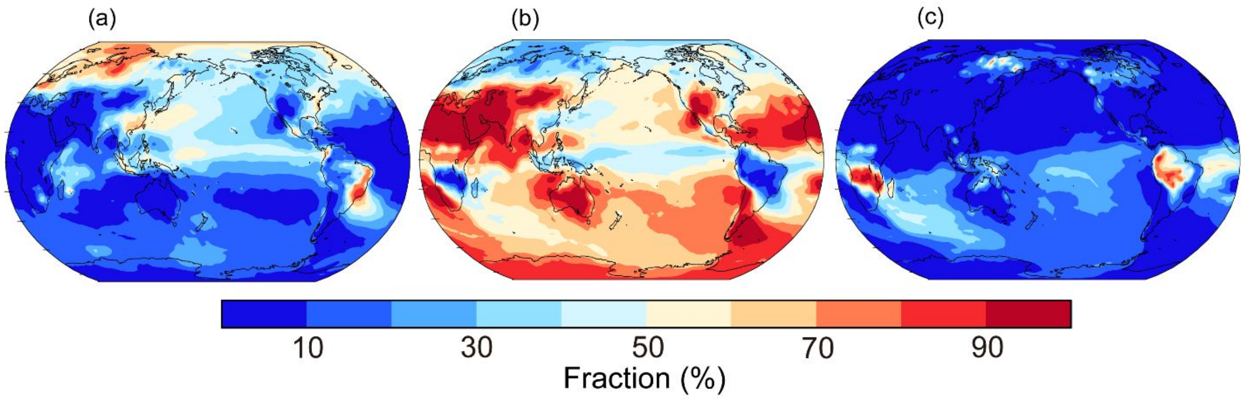

Figure S7. Percentage contribution of bioaccessible Fe deposition fluxes from (a) anthropogenic (15% and 15%), (b) lithogenic (78% and 72%), and (c) pyrogenic (7% and 12%) from the simulation with the low estimate of smelting Fe emission factors. The parentheses represent contribution of bioaccessible Fe deposition fluxes to the global ocean and the Southern Ocean (> 60°S), respectively.

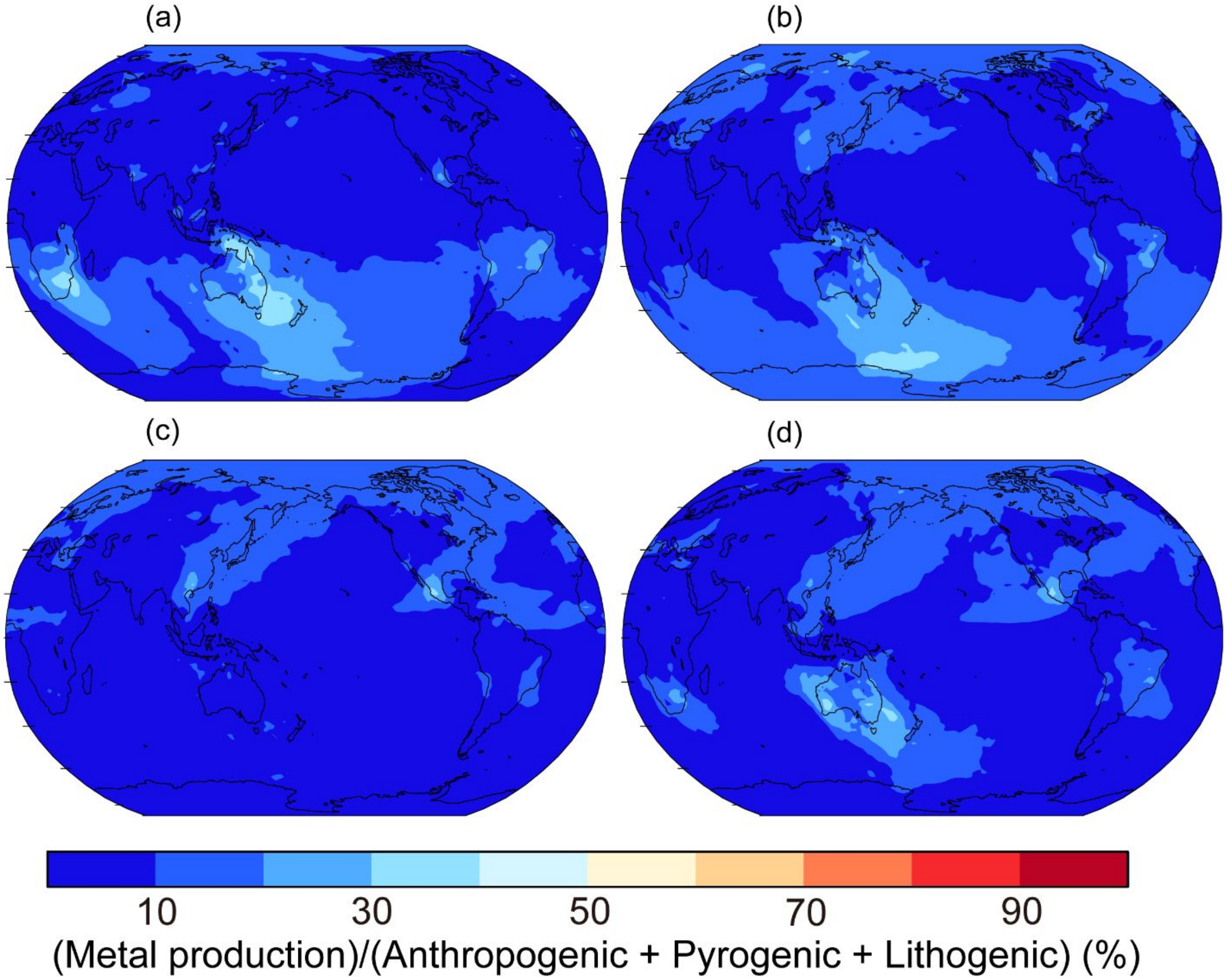

Figure S8. Percentage contribution of metal production to bioaccessible Fe deposition fluxes (%) for (a) austral fall (4% and 11%), (b) winter (1% and 14%), (c) spring (3% and 5%), and (d) summer (3% and 6%) from the simulation with the low estimate of smelting Fe emission factors (austral fall: March, April, May; winter: June, July, August; spring: September, October, November; summer: December, January, February). The color represents the (metal production)/(anthropogenic + pyrogenic + lithogenic) ratio (percent). The parentheses represent averaged contribution of metal production with the low estimate to the global ocean and the Southern Ocean (> 60°S), respectively.

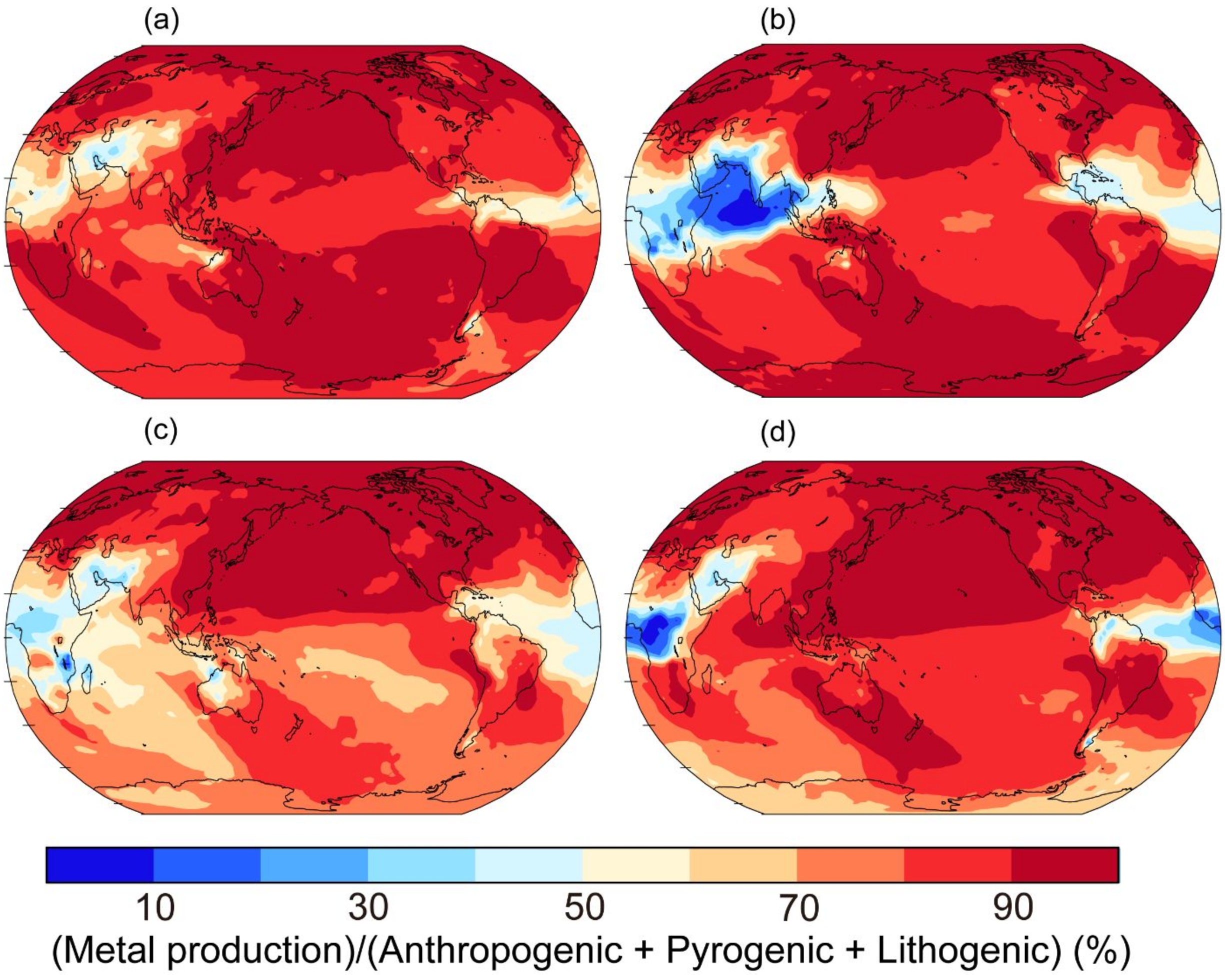

Figure S9. Percentage contribution of metal production to bioaccessible Fe deposition fluxes (%) for (a) austral fall (82% and 88%), (b) winter (55% and 91%), (c) spring (71% and 78%), and (d) summer (96% and 100%) from the simulation with the high estimate of smelting Fe emission factors (austral fall: March, April, May; winter: June, July, August; spring: September, October, November; summer: December, January, February). The color represents the (metal production)/(anthropogenic + pyrogenic + lithogenic) ratio (percent). The parentheses represent averaged contribution of metal production with the high estimate to the global ocean and the Southern Ocean (> 60°S), respectively.

Table S1. Annual Fe emissions from anthropogenic sources in different global studies.

study	metal production, fossil fuel, and biofuel combustion (Tg yr^{-1})	
	sub-micron	super-micron
low case	0.31 (11%)	1.1 (18%)
central case	1.1 (76%)	1.5 (39%)
high case	4.1 (93%)	3.3 (71%)
ref (11)	0.11	1.8
ref (26)	0.1	0.56
ref (27)	1.1 (65%)	1.1 (33%)

Note: The parentheses represent the contribution of metal production source to total Fe emission. Smelting sources include iron-ore sintering, pig-iron production, steel-making, aluminum (Al), copper (Cu), lead (Pb), and zinc (Zn) smelting [27]. Uncertainty calculations in the smelting Fe emission factors were performed using low, central, and high estimates of smelting Fe emission factors [27].

Table S2. Summary of the metal content of aerosols (%) used to estimate anthropogenic, pyrogenic, and lithogenic emissions of trace metals in the simulations.

sector	Fe in $PM_{2.5}$	Fe in PM_{10}	Si in $PM_{2.5}$
energy	4.18	4.20	15.79
industry	3.10	3.44	7.79
iron and steel industry	26.1	26.1	8.82
transportation	0.54	0.61	0.33
shipping	0.96	1.71	0.34
residential	0.18	0.04	0.48
waste	2.49	0.10	6.87
biofuel except iron and steel industry	0.01	0.01	0.03
aircraft	0.04	0.19	0.00
other fossil fuel combustion	1.40	1.40	5.84
agricultural fire	0.01	3.4	0.02
flaming fire in grassland	0.36	3.4	0.53
smoldering fire in grassland	0.02	3.4	0.22
flaming fire in forest	0.14	3.4	0.38
smoldering fire in forest	0.02	3.4	0.20
mineral dust	3.47	2.52	22.07

Note: The metal content of PM except Fe in iron and steel industry, shipping, and mineral dust was obtained from the compilation of source-specific measurements in fine particulate matter ($PM_{2.5}$) and coarse particulate matter (PM_{10}) [35, 46]. Fe content in PM from iron and steel industry was taken from the default model [11]. Global mean Fe content for mineral dust was calculated for fine particulate matter (bin1 and bin2) and coarse particulate matter (bin3 and bin4).

Table S3. Summary of Fe content in each mineral from the compilation of measurements [50].

Fe mineralogy	Fe content (wt %)
hematite	57.5
illite	4.0
smectite	2.6
kaolinite	0.2
feldspar	0.3
goethite	62.9
chlorite	13.3
vermiculite	6.7
calcite	0.0
quartz	0.0
mica	4.2
gypsum	0.0

Table S4. Summary of scaling factors of Fe content, which were used to estimate lithogenic emissions of Fe for East Asian dust from clay-sized soils in the simulations.

Fe species	scaling factor of Fe content
(1) Fe_{ox1}	19.25
(2) Fe_{ox2}	2.49
(3) Fe_{HCl}	1.80
(4) Fe_{R}	3.39

Note: Averaged Fe content for each Fe species in the clay-sized fraction of Chinese desert sediments [54] was used to scale that in East Asian clay-sized soils (35–50˚N, 70–120˚E) in the simulations. (1) Fe_{ox1} (ferrihydrite and lepidicrocite attached on Kaolinite and Feldspar), (2) Fe_{ox2} (hematite and goethite), (3) Fe_{HCl} (Fe-containing clay minerals such as illite, smectite, and chlorite), (4) Fe_{R} (residual Fe such as Vermiculite).

Table S5. Annual aerosol emissions from lithogenic sources and averaged Fe content by weight in different global studies.

study	dust emission (Tg yr^{-1})	Fe content (wt %)
this study	3597	2.59
ref (11)	5070	2.65
ref (29)	1614	3.50
ref (30)	1181	3.20
ref (31)	1767	3.22

Table S6. Comparison of bioaccessible Fe deposition flux (Gg Fe yr^{-1}) to the world ocean and the Southern Ocean between this study and previous studies.

study	metal production	combustion	lithogenic
world ocean			
low case	5	44	168
central case	113		
high case	548		
ref (11)		47	169
ref (29)			207
ref (30)		59	76
ref (31)		40	391
Southern Ocean (> 60°S)			
low case	0.06	0.15	0.53
central case	0.42		
high case	3.72		
ref (11)		0.06	0.11
ref (29)			0.17
ref (30)		0.09	0.24
ref (31)		0.19	0.32

Note: Uncertainty calculations in the smelting Fe emission factors were performed using low, central, and high estimates of smelting Fe emission factors [27]. Bioaccessible iron deposition flux from combustion is the sum of those from anthropogenic and pyrogenic sources except metal production.
